# Intra-abdominal hypertension and abdominal compartment syndrome in patients admitted to the ICU

**DOI:** 10.1186/s13613-020-00746-9

**Published:** 2020-10-01

**Authors:** Marije Smit, Bart Koopman, Willem Dieperink, Jan B. F. Hulscher, H. Sijbrand Hofker, Matijs van Meurs, Jan G. Zijlstra

**Affiliations:** 1 Department of Critical Care (BA 49), University Medical Center Groningen, University of Groningen, PO Box 30001, 9700 RB Groningen, The Netherlands; 2 Department of Pediatric Surgery, University Medical Center Groningen, University of Groningen, PO Box 30001, 9700 RB Groningen, The Netherlands; 3 Department of Surgery, University Medical Center Groningen, University of Groningen, PO Box 30001, 9700 RB Groningen, The Netherlands

**Keywords:** Intra-abdominal hypertension, Abdominal compartment syndrome, Intra-abdominal pressure, Decompressive laparotomy

## Abstract

**Background:**

Intra-abdominal hypertension is frequently present in critically ill patients and is an independent predictor for mortality. Risk factors for intra-abdominal hypertension and abdominal compartment syndrome have been widely investigated. However, data are lacking on prevalence and outcome in high-risk patients. Our objectives in this study were to investigate prevalence and outcome of intra-abdominal hypertension and abdominal compartment syndrome in high-risk patients in a prospective, observational, single-center cohort study.

**Results:**

Between March 2014 and March 2016, we included 503 patients, 307 males (61%) and 196 females (39%). Patients admitted to the intensive care unit with a diagnosis of pancreatitis, elective or emergency open abdominal aorta surgery, orthotopic liver transplantation, other elective or emergency major abdominal surgery and trauma were enrolled. One hundred and sixty four (33%) patients developed intra-abdominal hypertension and 18 (3.6%) patients developed abdominal compartment syndrome. Highest prevalence of abdominal compartment syndrome occurred in pancreatitis (57%) followed by orthotopic liver transplantation (7%) and abdominal aorta surgery (5%). Length of intensive care stay increased by a factor 4 in patients with intra-abdominal hypertension and a factor 9 in abdominal compartment syndrome, compared to patients with normal intra-abdominal pressure. Rate of renal replacement therapy was higher in abdominal compartment syndrome (38.9%) and intra-abdominal hypertension (8.2%) compared to patients with normal intra-abdominal pressure (1.2%). Both intensive care mortality and 90-day mortality were significantly higher in intra-abdominal hypertension (4.8% and 15.2%) and abdominal compartment syndrome (16.7% and 38.9%) compared to normal intra-abdominal pressure (1.2% and 7.1%). Body mass index (odds ratio 1.08, 95% confidence interval 1.03–1.13), mechanical ventilation at admission (OR 3.52, 95% CI 2.08–5.96) and Apache IV score (OR 1.03, 95% CI 1.02–1.04) were independent risk factors for the development of intra-abdominal hypertension or abdominal compartment syndrome.

**Conclusions:**

The prevalence of abdominal compartment syndrome was 3.6% and the prevalence of intra-abdominal hypertension was 33% in this cohort of high-risk patients. Morbidity and mortality increased when intra-abdominal hypertension or abdominal compartment syndrome was present. The patient most at risk of IAH or ACS in this high-risk cohort has a BMI > 30 kg/m^2^ and was admitted to the ICU after emergency abdominal surgery or with a diagnosis of pancreatitis.

## Background

Intra-abdominal hypertension (IAH) is frequently present in critically ill patients and is an independent predictor for mortality [1–3]. When IAH progresses to abdominal compartment syndrome (ACS), organ failure occurs by definition [4] and mortality is very high [5]. World Society of the Abdominal Compartment Syndrome (WSACS, currently WSACS—the Abdominal Compartment Society) guidelines recommend protocolized monitoring of intra-abdominal pressure (IAP) in high-risk patients every 4–6 h [4, 6] . However, 18–82% of physicians indicate they do not measure IAP [7, 8], so compliance with these guidelines may be improved. Up-to-date data regarding incidence and prognosis of IAH and ACS may further improve recognition of the patient at risk and, thus, contribute in optimization of monitoring and management. Better understanding of the risks associated with IAH is necessary to improve outcome [9].

Recently, data on prevalence of IAH and ACS were summarized by Padar et al. [5]. Data of 285 consecutive patients from a mixed medical–surgical intensive care unit (ICU) showed that ACS occurred in 3% of patients [3]. In a mixed multi-center ICU population of 491 consecutive patients, ACS occurred in 6% of patients. The difference between the studies was attributed to the different case mix [10].

Risk factors for IAH and ACS have been widely investigated and include a presenting diagnosis of pancreatitis, abdominal surgery, ileus, intra-abdominal infection and patients suffering severe trauma [11, 12]. In the largest study to date, Reintam Blaser et al. reported risk factors for IAH in 563 mechanically ventilated patients [13]. A body mass index (BMI) > 30 kg/m^2^, positive end-expiratory pressure (PEEP) > 10 cmH_2_O, a ratio of arterial oxygen partial pressure to fractional inspired oxygen (PaO2/FiO2) < 300, use of vasopressors/inotropes, pancreatitis, hepatic failure/cirrhosis with ascites, gastro-intestinal (GI) bleeding and laparotomy on admission day were all risk factors for IAH. It is noteworthy that only ICU mortality was reported as an outcome parameter.

We performed the current study to gain more insight in the prevalence and outcome of IAH and ACS in high-risk ICU patients. Our objectives were first to investigate the prevalence of IAH and ACS in a cohort of high-risk patients. Second, we investigated the morbidity by recording incidence of renal replacement therapy, duration of mechanical ventilation, length of ICU stay and management of ACS. Third, we recorded ICU mortality and 90-day mortality in this cohort. Our hypotheses were that in a cohort of high-risk patients, prevalence of IAH and ACS would be higher than reported in the literature for consecutive patients admitted to the ICU and that morbidity and mortality would be increased in IAH and ACS, as reported in other cohorts. Furthermore, we hypothesized that further identification of the patient most at risk of IAH and ACS in a high-risk cohort would be possible.

## Methods

In this prospective, observational, single-center cohort study, we enrolled consecutive adult patients with known risk factors for primary IAH, admitted to the ICU of a tertiary academic teaching hospital. Admission type (elective abdominal surgery, emergency abdominal surgery and non-surgical) and two main diagnoses were recorded at admission.

Inclusion criteria were: a main diagnosis of pancreatitis, elective or emergency open abdominal aorta surgery, orthotopic liver transplantation (OLT), other elective or emergency major abdominal surgery and trauma with either abdominal injury or with a combination of chest and pelvic injury (since the latter patients have a high risk of concomitant abdominal injury). Exclusion criteria were: age < 18 years and contraindications for urine catheter placement.

IAP was measured directly after admission to the ICU and subsequently every 4 h for seven days or until discharge from the ICU. IAP was measured according to a standardized protocol using 25 ml of sterile saline as priming volume with the symphysis pubis as the reference point. Patients were in supine position during IAP measurement. If IAP measurement could not be performed in supine position for reasons of patient care, head-of-bed elevation up to 30 degrees was accepted. If IAP was ≥ 20 mmHg at any time, the IAP measurement was repeated after 1 h. If ACS was diagnosed (see Table 1 for definitions of IAH and ACS), it was left up to the attending physician whether or not an intervention (medical, interventional radiology or surgical) was performed. Possible interventions were based on the WSACS recommendations for ACS management [4]. Medical management included a temporary stop in enteral feeding, insertion of a nasogastric tube and drainage of stomach contents, increase in sedation and/or analgesia, administration of neuromuscular blockers, placement of rectal cannula or rectal enema and fluid removal using diuretics or renal replacement therapy (RRT). RRT consisted of continuous veno-venous hemofiltration (CVVH) or haemodialysis. Interventional radiology management included drainage of ascites or other abdominal fluid collections and surgical management included surgical decompression. The aim was to perform surgical decompression by an experienced abdominal surgeon within 6 h of failure of medical and interventional radiology management in ACS. If the patient showed no signs of clinical improvement, in the opinion of the attending physician, the study continued for longer than seven days until patient status improved.

**Table 1:** Definitions of IAH, IAH grades and ACS

	Definition
Intra-abdominal Hypertension (IAH)	Sustained intra-abdominal pressure (IAP) ≥ 12 mmHg^a^
IAH grade I	IAP 12–15 mmHg^a^
IAH grade II	IAP 16–20 mmHg^a^
IAH grade III	IAP 21–25 mmHg^a^
IAH grade IV	IAP > 25 mmHg^a^
IAH at admission	First and second IAP measurements after admission were ≥ 12 mmHg
Abdominal Compartment Syndrome (ACS)	Sustained IAP > 20 mmHg associated with new organ dysfunction or failure^a^

^a^[4]

### Definitions

See Table 1 for definitions of IAH, IAH grades and ACS.

The Institutional Review Board of our hospital approved the study (METc 2013/123).

### Statistical analysis

Statistical analysis was performed using IBM SPSS Statistics 22. For continuous variables, a one-way analysis of variance was performed to calculate the difference between the groups of normal IAP, IAH and ACS and between the groups of elective abdominal surgery, emergency abdominal surgery and non-surgical patients. Chi-square tests for independence were performed for categorical variables. A binary logistic regression analysis was performed to analyze whether risk factors for IAH or ACS that were significant in a univariate analysis were independent.

## Results

### Demographics

**Fig. 1:**
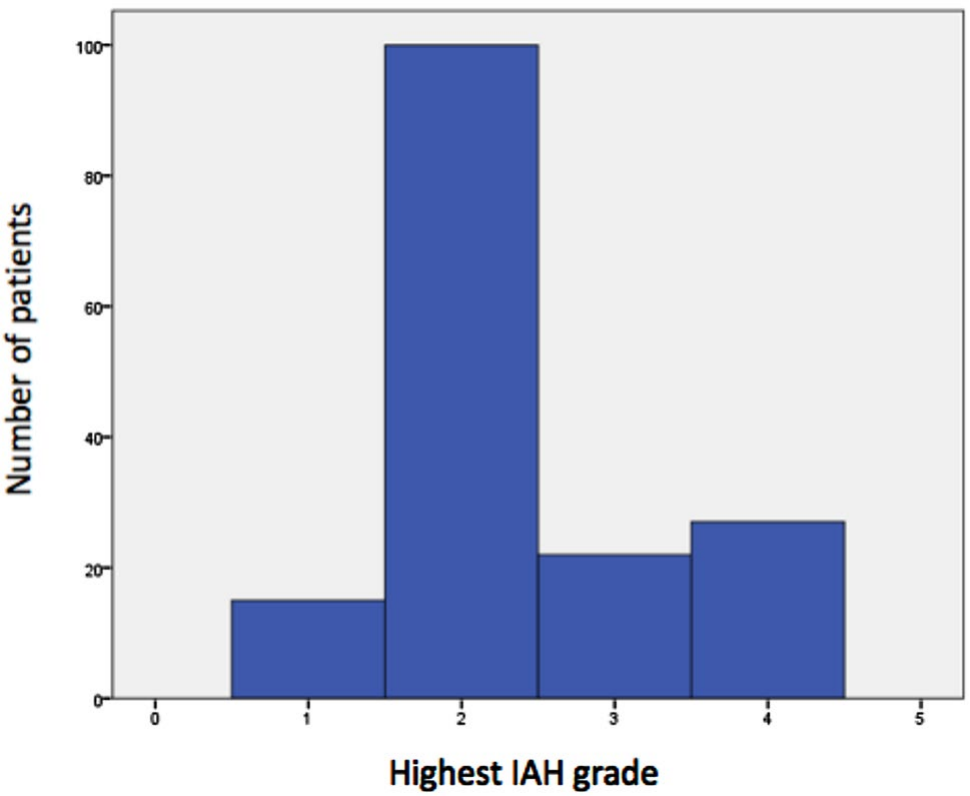
Highest IAH grade in patients with IAH or ACS (*N* = 164)

During study enrollment from March 3, 2014 until March 31, 2016 503, patients were included. In the study period, a total of 7703 patients were admitted to the ICU in our hospital. IAP was measured 5908 times, 127 times (2%) with head-of-bed elevation up to 30 degrees. Three hundred and thirty nine patients were included after elective surgery (67%), 120 patients after emergency surgery (24%) and 44 non-surgical patients (9%) were included (Table 2). Mean IAP at ICU admission was 11.1 mm Hg (SD 5.5). In 212 patients (42%), IAP was ≥ 12 mm Hg at admission (Additional file 1: Figure S1). During ICU stay, IAH (a sustained IAP ≥ 12 mm Hg) developed in 164 patients (33%) and 18 patients (3.6%) developed ACS. In the 164 patients with IAH, 111 presented with this diagnosis at admission (67.7%). Most IAH patients had grade II IAH (Fig. 1).

**Table 2:** Demographics by IAP category

	Total	Normal IAP	IAH, no ACS	ACS	*p* value
Total number of patients (%)	503 (100)	339 (67.4)	146 (29.0)	18 (3.6)	
Male gender (%)	307 (61.0)	204 (60.2)	93 (63.7)	10 (55.6)	0.681
Age in years, mean (SD)	62.2 (12.9)	62.6 (12.3)	61.6 (13.5)	60.6 (17.5)	0.726
Body Mass Index in kg/m^2^, mean (SD)	26.3 (5.0)	25.9 (4.8)	27.1 (5.3)	27.7 (6.0)	0.025
Admission type					
Elective surgery (%)	339 (100)	278 (82.0)	58 (17.1)	3 (0.88)	< 0.001
Emergency surgery (%)	120 (100)	52 (43.3)	60 (50.0)	8 (6.7)	
Non-surgical (%)	44 (100)	9 (20.5)	28 (63.6)	7 (15.9)	
Apache IV score mean (SD) (*n* = 489)	54.2 (22.1)	47.8 (18.6)	66.2 (21.4)	82.8 (26.8)	< 0.001
SAPS II score mean (SD)	33.1 (14.1)	28.6 (12.1)	41.3 (12.6)	52.0 (15.9)	< 0.001
Mechanical ventilation at admission (%)	299 (59.4)	163 (48.1)	120 (82.2)	16 (88.9)	< 0.001

*IAP* intra-abdominal pressure, *IAH* Intra-abdominal hypertension, *ACS* Abdominal Compartment Syndrome

Table 3 shows admission diagnosis by IAP group for the elective abdominal surgery, the emergency abdominal surgery and the non-surgical management groups. In the elective abdominal surgery group, 18.0% of patients developed IAH or ACS and in the emergency abdominal surgery group, 56.7% developed IAH or ACS. Thirty five of 44 patients in the non-surgical management group developed IAH or ACS (79.5%). However, 12 patients in this group had emergency abdominal surgery within 24 h of admission to the ICU, but were analyzed in the non-surgical group. Ten patients (83%) developed IAH or ACS. The most prevalent diagnoses in the remaining 32 patients were pancreatitis in 7 patients and trauma in 7 patients.

**Table 3:** Admission diagnosis by IAP group and admission type

Admission type	Total number of patients	Normal IAP	IAH, no ACS	ACS
Admission diagnosis				
**Elective surgery (%)**	**339**	**278 (82.0)**	**58 (17.1)**	**3 (0.9)**
Abdominal aorta	45	30	13	2
Cancer other GI tract (hepatoma, gallbladder etc.)	100	85	14	1
Cancer colon/rectum	79	65	14	0
Oesophageal cancer	29	25	4	0
Stomach cancer	15	13	2	0
Small intestinal cancer	10	9	1	0
Pancreatic cancer	25	21	4	0
Trauma	2	1	1	0
Other	34	29	5	0
**Emergency surgery (%)**	**120**	**52 (43.3)**	**60(50.0)**	**8(6.7)**
Orthotopic liver transplantation (%)	42	17(40.5)	22(52.4)	3(7.1)
Abdominal aorta	13	5	7	1
Complications of previous GI surgery (anastomotic leakage, bleeding, abscess, infection)	13	8	4	1
GI perforation/rupture	11	6	4	1
GI vascular ischemia	8	3	5	0
GI obstruction	3	0	3	0
GI hemorrhage	4	2	1	1
Trauma	6	3	3	0
Other	20	8	11	1
**Non-surgical management (%)**	**44**	**9(20.5)**	**28(63.6)**	**7(15.9)**
1. No surgery	32	7	19	6
Pancreatitis	7	1	2	4
Trauma	7	3	4	0
GI/intra-/retroperitoneal hemorrhage	2	0	1	1
GI sepsis	6	0	5	1
Other	10	3	7	0
2. Emergency abdominal surgery within 24 h of ICU admission	12	2	9	1
Trauma	2	0	2	0
GI sepsis	4	2	1	1
GI perforation/rupture	3	0	3	0
GI obstruction	1	0	1	0
GI ischemia	1	0	1	0
Cholecystitis	1	0	1	0

*IAP* intra-abdominal pressure, *IAH* intra-abdominal hypertension, *ACS* Abdominal Compartment Syndrome, *GI* gastro-intestinal

IAH or ACS developed in 6/7 patients with pancreatitis (85.7%), in 25/42 patients after OLT (59.5%) and in 23/58 patients after aorta surgery (39.7%). Highest prevalences of ACS were observed in pancreatitis (57.1%), followed by OLT (7.1%) and elective or emergency aorta surgery (5.2%). Although none of the 17 trauma patients developed ACS, 10/17 patients developed IAH (58.8%).

Table 4 summarizes risk factors for IAH and ACS by admission type. Mean BMI was not different between the groups of elective abdominal surgery, emergency abdominal surgery and non-surgical patients (*p* = 0.77). Apache IV scores and number of patients with IAH and ACS was significantly different between all 3 groups (*p* < 0.01). Mechanical ventilation at admission was significantly different between the elective abdominal surgery group and the emergency abdominal surgery group (*p* < 0.01) and between the elective abdominal surgery group and non-surgical group (*p* < 0.01).

**Table 4:** Demographics by admission type

	Total	Elective abdominal surgery	Emergency abdominal surgery	Non-surgical	*p* value
Total number of patients	503	339	120	44	
Number of patients with IAH and ACS (%)	164 (33)	61 (18)	68 (57)	35 (80)	< 0.01*
BMI mean (SD)	26.3 (5.0)	26.3 (4.8)	26.5 (5.6)	25.9 (5.2)	0.77
Apache IV score mean (SD)	54.2 (22.1)	46.5 (13.6)	68.4 (27.1)	78.2 (26.5)	< 0.01*
Mechanical ventilation at admission (%)	299 (59.4)	167 (49)	100 (83)	32 (73)	< 0.01**

^*^Significant difference between all 3 groups

^**^Mechanical ventilation at admission was significantly different between the elective abdominal surgery group and the emergency abdominal surgery group and between the elective abdominal surgery group and the non-surgical group only

In a binary logistic regression analysis BMI [odds ratio (OR) 1.08, 95% confidence interval (CI) 1.03–1.13], Apache IV score (OR 1.03, 95% CI 1.02–1.04), admission after emergency surgery (OR 2.8, 95% CI 1.61–4.87) and mechanical ventilation at admission (OR 3.52, 95% CI 2.08–5.96) were independent risk factors for development of IAH or ACS in this cohort (Table 5). The odds ratios for IAH or ACS in this study were increased in emergency abdominal surgery (OR 2.80, 95% CI 1.61–4.87) and in non-surgical patients (OR 8.90, 95% CI 3.62–21.90) compared to elective abdominal surgery patients.

**Table 5:** Binary logistic regression of risk factors for IAH or ACS (n = 489)

	*p* value	Odds ratio	95% confidence interval
BMI (per unit)	< 0.01	1.08	1.03–1.13
Admission type			
Elective abdominal surgery	< 0.01		
Emergency abdominal surgery	< 0.01	2.80	1.61–4.87
Non-surgical	< 0.01	8.90	3.62–21.90
Apache IV score (per unit)	< 0.01	1.03	1.02–1.04
Mechanical ventilation at admission (yes/no)	< 0.01	3.52	2.08–5.96

*IAH* intra-abdominal hypertension, *ACS* Abdominal Compartment Syndrome, *BMI* Body Mass Index

^*^The odds ratio for IAH or ACS was 2.8 in emergency abdominal surgery compared to elective abdominal surgery and 8.9 in the non-surgical group compared to elective abdominal surgery

### ACS management

Management of ACS was medical or radiological in 12/18 (66.7%) patients and surgical in 6/18 (33.3%) patients (Table 6). Medical management included a temporary stop in enteral feeding (*n* = 1), suction of nasogastric tube (*n* = 4), additional analgesia (*n* = 5), placement of a rectal cannula (*n* = 2), rectal enema (*n* = 4) and/or fluid removal using diuretics or dialysis (*n* = 2). Radiological management included ascites drainage (*n* = 2). Surgical management included relaparotomy and primary abdominal closure in 3 OLT patients. The cause of ACS in these cases was peri-operative hemorrhage and coagulopathy. In 3 other patients, decompressive laparotomy was performed with open abdomen management. The diagnoses in these patients were pancreatitis (*n* = 1), GI sepsis (*n* = 1) and hilar cholangiocarcinoma (*n* = 1) for which an elective extended right hemi-hepatectomy had been performed. The open abdomen was managed with a negative pressure commercial vacuum system (KCI Abthera, GD medical, Eindhoven, Netherlands) in all patients. These 3 patients died in the ICU with multiple organ failure. The patients who died had higher Apache IV scores (97 vs. 83 in all ACS patients) and required RRT more often than those who survived (100% vs. 39% in all ACS patients). Decompressive laparotomy and open abdomen management were delayed in the patient with GI sepsis. Primary closure of the abdomen took place during relaparotomy after ACS had been diagnosed and decompressive laparotomy with open abdomen was performed one day later due to on-going hemodynamic instability.

**Table 6:** Admission diagnosis and management in ACS patients (*n* = 18) by admission type

Admission typ**e**	Number of patients	Management of ACS	Number of patients	Surgery type	Number of patients	ICU mortality
Admission diagnosis						
**Elective surgery**	**3**					**1**
Aorta	2	Medical	2			0
Cancer other GI tract^*^	1	Surgical	1	Decompression + open abdomen	1	1
**Emergency surgery**	**8**					**0**
Orthotopic liver transplant	3	Surgical	3	Relaparotomy + primary closure	3	0
Aorta	1	Medical	1			0
Hemorrhage lower GI tract	1	Medical	1			0
GI perforation	1	Medical	1			0
Complications of previous GI surgery^**^	1	Medical	1			0
GI surgery other^***^	1	Medical	1			0
**Non-surgical**	**7**					**2**
Pancreatitis	4	Medical Radiological + Surgical	3 1	Decompression + open abdomen	1	0 1
GI sepsis	2	Radiological Surgical	1 1	Decompression + open abdomen	1	0 1
Hemorrhage intra-/retroperitoneal	1	Medical	1			0

*ACS* Abdominal Compartment Syndrome, *ICU* Intensive Care Unit, *GI* gastro-intestinal

^*^Diagnosis: Extended right hemi-hepatectomy with extra-hepatic bile duct resection and liver segment 1 resection due to hilar cholangiocarcinoma, Klatskin Bismuth type 3a/4

^**^Diagnosis upon relaparotomy: Perforation small intestine after correction hernia cicatricalis

^***^Laparotomy for fulminant pancolitis due to clostridium difficile infection

### Morbidity and mortality

The rate of RRT was significantly higher in IAH and ACS compared to the normal IAP group (8.2% and 38.9% vs. 1.2%) (*p* < 0.01) and duration of mechanical ventilation was longer (91.8 and 196.3 h vs. 14.0 h) (*p* < 0.01). Length of ICU stay was longer in IAH and ACS compared to the normal IAP group (6.1 and 12.0 days vs. 1.3 days) (*p* < 0.01). ICU mortality was significantly higher in the IAH and ACS group compared to the normal IAP group (4.8% and 16.7% vs. 1.2%) (*p* < 0.01). 90 day mortality was higher in IAH and ACS compared to the normal IAP group (15.2% and 38.9% vs. 7.1%) (*p* < 0.01) (Table 7).

**Table 7:** Morbidity and mortality of patients with IAH and ACS compared to patients with normal IAP

	Total group	Normal IAP	IAH, no ACS	ACS	*p* value
Total number of patients (%)	503 (100)	339 (67.4)	146 (29.0)	18 (3.6)	
Length of ICU stay in days, mean (SD)	3.1 (7.1)	1.3 (1.8)	6.1 (10.5)	12.0 (15.9)	< 0.01
Renal Replacement Therapy (%)	23 (4.6)	4 (1.2)	12 (8.2)	7 (38.9)	< 0.01
Mechanical ventilation duration in hours, mean (SD)	55.0 (155.4)	14.0 (32.9)	91.8 (194.7)	196.3 (341.6)	< 0.01
ICU mortality (%)	14 (2.8)	4 (1.2)	7 (4.8)	3 (16.7)	< 0.01
90 day mortality (%)	53 (10.6)	24 (7.1)	22 (15.2)	7 (38.9)	< 0.01

*IAH* intra-abdominal hypertension, *ACS* Abdominal Compartment Syndrome, *IAP* intra-abdominal pressure

## Discussion

This prospective study demonstrates an overall ACS incidence of 3.6% in a cohort with a high estimated a priori risk. The incidence of IAH in our study was 33%. Compared to the literature, the incidence of ACS in this cohort was low.

In a study of 83 patients published in 2008, the incidence of ACS was 12% in a heterogeneous intensive care population [14]. The incidence of ACS in the current study is comparable to a recent study in a mixed medical–surgical ICU where ACS occurred in 3% of patients, although this study included consecutive ICU patients who were not selected for their high-risk for IAH or ACS [3]. This might be explained by the fact that 67% of the patients in the current study were admitted after elective abdominal surgery. The prevalence of IAH was relatively low in this group (18%) compared with admission after emergency abdominal surgery (57%) and the non-surgical admission group (80%).

Highest prevalence of ACS was observed in patients with pancreatitis, OLT and abdominal aorta surgery. Older studies report an incidence of ACS in pancreatitis of 25–56% and of 33–41% after major abdominal surgery [15–17]. The incidence of ACS after OLT was 31% in a study of 108 patients in 2003.[18]. The low prevalence of ACS in our cohort may be due to improved peri-operative and intensive care management including restrictive peri-operative fluid management and meticulous haemostasis. Furthermore, ACS might be decreasing through early recognition and management of IAH [9].

Six of the 7 patients diagnosed with pancreatitis had IAH (86%) and 4 had ACS (57%). These high numbers corroborate the findings of our earlier retrospective cohort study which found that IAH and ACS are common in patients with severe acute pancreatitis. This study led to the implementation of an IAP monitoring protocol in our ICU and called for national and international guidelines on pancreatitis to be updated to include IAP monitoring as standard of care. Currently, IAP is measured every 4 h in patients at risk of IAH (including those with pancreatitis) in our ICU [19]. The prevalence of ACS is higher than described in a 2014 review, where prevalences of IAH and ACS in pancreatitis were 54–66% and 22–38%, respectively [20]. This discrepancy may be explained by the fact that there were only 7 patients with pancreatitis in the study. Moreover, a selection of patients with pancreatitis has occurred since our hospital is a tertiary referral center for patients with pancreatitis and patients are usually referred to our hospital when an intervention is imminent or when complications occur. IAH can deteriorate by aggressive fluid resuscitation [21]. Restrictive fluid management strategies such as recommended by Aggarwal et al. were not applied in the group of patients with pancreatitis and this may have contributed to the development of IAH and ACS [21]. None of the 17 trauma patients in this study developed ACS. However, the incidence of IAH was 10/17 (58.8%). These findings corroborate a study in 81 trauma patients where the incidence of IAH was 75% and no patient developed ACS [22]. We, therefore, share the authors’ conclusion that the attenuation of ACS to the less deleterious IAH might be considered a success of development in trauma- and critical care.

In the “Incidence, Risk Factors, and Outcomes of Intra-abdominal Hypertension in Critically Ill Patients”(IROI) Study [10], 491 consecutive patients from 15 ICUs worldwide were included. Comparable to our study, elective surgery patients and a mixture of mechanically ventilated and spontaneously breathing patients were included. IAH was present in 34% on admission day and in 48.9% of patients during the observation period. IAH was observed in 99 medical patients (53.2%), in 86 emergency surgery patients (56.6%) and in 55 elective surgery patients (35.9%). ACS was noted in 6.3% of patients. IAH grade II was most prevalent in IAH patients in our study. This is contrary to findings in other studies including the IROI study where IAH grade I is most prevalent [10, 13]. Differences in prevalence of IAH and ACS between the studies are probably due to the selection of patients. When the high-risk patients in our study developed IAH, the IAH grade was higher than in studies with consecutive ICU patients. This may be indicative of their higher risk in developing IAH and ACS. Almost 68% of the patients with IAH or ACS were diagnosed at admission to the ICU. This is in line with other studies where IAH developed after day 1 in only 31–34% of patients [3, 10, 14]. IAP was ≥ 12 mmHg at admission in 42% of patients. This may have been due to agitation and/or pain immediately post-surgery or post-transportation to the ICU. The rapid decrease in IAP may be explained by adequate patient care in the ICU, including pain relief. Morbidity and mortality increased in the IAH and ACS group compared to the normal IAP group. This observation is in line with the literature where IAH has been found to be an independent predictor for ICU mortality [2]. Oliguria and renal dysfunction are among the earliest signs of increasing IAP [23] and IAP is an independent cause of renal impairment [16]. We found that the rate of RRT was significantly higher in IAH and ACS (8.2% and 38.9%) compared to the normal IAP group (1.2%).

In 2004, Malbrain found that BMI was the only independent risk factor for IAH development [24]. In the IROI study, BMI, APACHE II score ≥ 18, PEEP > 7 cm H_2_O, presence of abdominal distension and absence of bowel sounds were associated with IAH [10]. In a binary logistic regression analysis of risk factors for IAH or ACS in the current study, BMI, Apache IV score and ventilation at admission were independent risk factors. Murphy et al. found that admission type (medical vs surgical vs trauma) was not a predictor of IAH [3]. Contrary to these findings, the odds ratios of IAH or ACS in this study were increased in emergency abdominal surgery (by 2.8) and in non-surgical patients (by 8.9) compared to elective abdominal surgery patients. However, these odds ratios should be interpreted with caution. It is important to note that there were only 44 patients in the non-surgical group (less than 9% of the total number of patients in the study), 12 of these patients had emergency abdominal surgery within 24 h of admission to the ICU and were analyzed in the non-surgical group and 10/12 developed IAH/ACS. Based on these data, it is our conclusion that there is an increased risk of IAH or ACS in emergency abdominal surgery compared to elective abdominal surgery and in patients with a diagnosis of pancreatitis, since this diagnosis was most prevalent among the remaining 32 patients in the non-surgical group with an IAH/ACS prevalence of 85.7%. Therefore, the patient most at risk of IAH and ACS in this high-risk cohort is the patient with a BMI > 30 kg/m^2^ who was admitted to the ICU after emergency abdominal surgery or with a diagnosis of pancreatitis.

In this observational study in a high-risk cohort of patients, ACS management strategies were not standardized. They consisted of medical, interventional radiology and surgical or combined approaches. Most patients were managed without decompressive laparotomy. These patients all survived their ICU stay. This illustrates further that non-surgical management is an important treatment option in critically ill patients with raised IAP [25]. When decompressive laparotomy and open abdomen management were performed, all patients died with multiple organ failure in the ICU. Although our study was not designed for treatment effects and the numbers are too small for conclusions, this mortality rate is higher than in the literature, where mortality after surgical decompression varies between 18 and 49% [26–28]. Possible explanations for the high ICU mortality in this small group of patients, other than coincidence, may be that, illustrated by their Apache IV scores, these patients were even more seriously ill than the other ACS patients and a delay in decompressive laparotomy that occurred in one of the patients. To date, there have been no interventional studies to answer the important question of which ACS patients need medical, radiological or surgical management. Therefore, the position and timing of decompressive laparotomy in ACS are still unknown [28].

The consequence of the heterogeneous patient population at risk for ACS in combination with the low incidence of ACS is that interventional studies can only be performed in large multi-center trials. A multi-center trial which randomizes between early decompressive surgery with open abdomen management and medical management in ACS patients might answer some of the many remaining questions. To base open abdomen management on sufficient evidence, a laparostomy registry entitled Open Abdomen Route has been implemented in 2015 [29]. An interim analysis shows that in 649 patients with open abdomen management, the indication to open the abdomen was postoperative ACS in 19 patients (2.9%) and pancreatitis in 37 patients (5.7%) [30]. For the near future, until more data are available, treatment of this life threatening syndrome will depend on sound clinical judgment and close cooperation between the critical care, gastro-enterology and surgical disciplines.

Although we have chosen to analyze the patients in this study in 3 groups: those with normal IAP, those with IAH and those with ACS, our data illustrate that IAH and ACS are a disease continuum, with ACS being the most serious. Early recognition and management of IAH may be key in preventing the occurrence of ACS with its increased morbidity and mortality. In our opinion, to recognize and manage IAH and ACS early, it is crucial that routine IAP measurement is performed in high-risk patients, especially in patients with pancreatitis and patients after emergency abdominal surgery.

### Strengths and limitations of this study

Our study is the largest prospective study to date that has investigated prevalence and a variety of outcome parameters of IAH and ACS in high-risk ICU patients. However, this was a single-center study in a tertiary hospital which may limit the applicability of the data.

A standardized approach to IAP measurement is important to ensure reproducibility [6]. In this study, IAP was consistently measured using the symphysis pubis as reference point instead of the mid-axillary line as advised in recent guidelines [4]. Since IAP measured with the mid-axillary line as reference point is significantly higher than the symphysis pubis in the supine position [31], an underestimation of IAP may have occurred in this study and, therefore, an underestimation of the number of patients with IAH and ACS. IAP should be measured in supine position [4]. In this study, head-of-bed elevation up to 30 degrees was accepted if IAP could not be performed in supine position for reasons of patient care. This occurred in 2% of IAP measurements. Since clinically relevant changes in IAP occur at head-of-bed increases above 20 degrees [32], head-of-bed elevation may have increased IAP in this minority of patients. The diagnoses in our study were based on two main diagnoses recorded at admission and analysis was performed on this data. Not all relevant information may have been available at the time of admission. Furthermore, since we did not measure IAP continuously, the exact duration of IAH cannot be concluded from the current study.

## Conclusions

The prevalence of ACS in a high-risk ICU population was 3.6% and the prevalence of IAH was 33%. Highest prevalence of ACS occurred in subgroups of patients with pancreatitis (57%), followed by orthotopic liver transplantation (7%) and elective or emergency abdominal aorta surgery (5%). Morbidity and mortality increased when IAH or ACS was present. The patient most at risk of IAH or ACS in this high-risk cohort has a BMI > 30 kg/m^2^ and was admitted to the ICU after emergency abdominal surgery or with a diagnosis of pancreatitis.

## Supplementary information

**Additional file 1: Figure S1.** IAP distribution at admission.

## Data Availability

The datasets used and/or analyzed during the current study are available from the corresponding author on reasonable request.

## Abbreviations

ACS: Abdominal compartment syndrome
Apache score: Acute physiology and chronic health evaluation score
BMI: Body mass index
CI: Confidence interval
CVVH: Continuous veno-venous hemofiltration
GI: Gastro-intestinal
IAH: Intra-abdominal hypertension
IAP: Intra-abdominal pressure
ICU: Intensive care unit
METc: Medische ethische toetsingscommissie (Institutional Review Board)
OLT: Orthotopic liver transplantation
OR: Odds ratio
PaO2/FiO2 ratio: Ratio of arterial oxygen partial pressure to fractional inspired oxygen
PEEP: Positive end-expiratory pressure
RRT: Renal replacement therapy
WSACS: World Society of the abdominal compartment syndrome (currently WSACS, the abdominal compartment society)
