# Supplementary figures and images for: Intra-abdominal hypertension and abdominal compartment syndrome in patients admitted to the ICU
Source: Ann Intensive Care. 2020 Oct 1;10:130. doi: 10.1186/s13613-020-00746-9 (PMC7530150; doi:10.1186/s13613-020-00746-9)

**Additional file 1: Figure S1**

Intra-abdominal pressure (IAP) distribution at admission

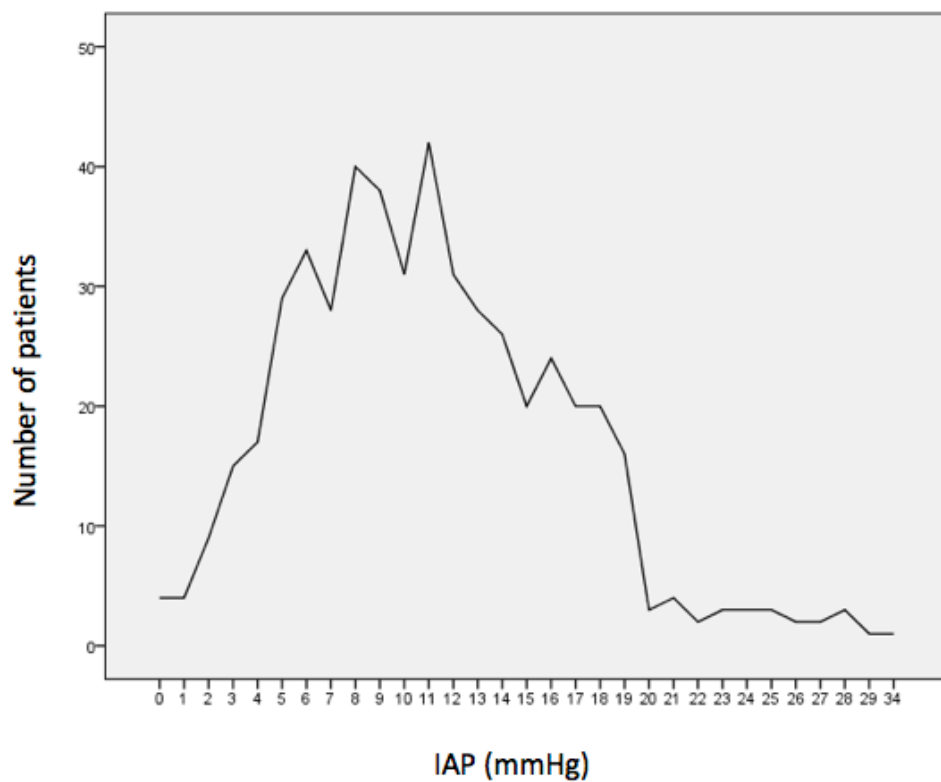

Supplement: Supplementary file 1 — Additional file 1: Figure S1. IAP distribution at admission. [file 13613_2020_746_MOESM1_ESM.pdf]
